# Role of Chondroitin Sulfation Following Spinal Cord Injury

**DOI:** 10.3389/fncel.2020.00208

**Published:** 2020-08-05

**Authors:** Rowan K. Hussein, Caitlin P. Mencio, Yasuhiro Katagiri, Alexis M. Brake, Herbert M. Geller

**Affiliations:** Laboratory of Developmental Neurobiology, Cell and Developmental Biology Center, National Heart, Lung, and Blood Institute, US National Institutes of Health, Bethesda, MD, United States

**Keywords:** proteoglycan, glycosaminoglycan, axon guidance, receptor tyrosine phosphatase, glial scar

## Abstract

Traumatic spinal cord injury produces long-term neurological damage, and presents a significant public health problem with nearly 18,000 new cases per year in the U.S. The injury results in both acute and chronic changes in the spinal cord, ultimately resulting in the production of a glial scar, consisting of multiple cells including fibroblasts, macrophages, microglia, and reactive astrocytes. Within the scar, there is an accumulation of extracellular matrix (ECM) molecules—primarily tenascins and chondroitin sulfate proteoglycans (CSPGs)—which are considered to be inhibitory to axonal regeneration. In this review article, we discuss the role of CSPGs in the injury response, especially how sulfated glycosaminoglycan (GAG) chains act to inhibit plasticity and regeneration. This includes how sulfation of GAG chains influences their biological activity and interactions with potential receptors. Comprehending the role of CSPGs in the inhibitory properties of the glial scar provides critical knowledge in the much-needed production of new therapies.

Traumatic injury to the adult spinal cord is a major public health problem, with approximately 18,000 new cases per year in the United States. The most frequent cause (39%) is automobile crashes, while falls account for about 32% of injuries. Virtually all spinal cord injured patients have long-term neurological damage, with less than 1% experiencing neurological recovery upon hospital discharge. This has resulted in nearly 300,000 people with spinal cord injury in the US alone. At present, despite a large amount of research, there are no approved therapies to treat spinal cord injury (SCI), likely because of our incomplete understanding of the pathophysiology of the condition. In this review article, we present the current evidence supporting a role for the sulfated glycosaminoglycan (GAG) chains of chondroitin sulfate proteoglycans (CSPGs) in acute and chronic spinal cord injuries, and how we might harness this knowledge to promote recovery of function.

Traumatic SCI occurs in two phases. The primary injury is a result of a mechanical distortion of the vertebral column which produces damage to axons at the site of injury. This results in severing both ascending and descending fiber tracts at the site of injury. The severing of the corticospinal tract and breakdown of the myelin sheath results in a loss of connectivity with cell bodies in the motor cortex and paralysis of dermatomes distal to the lesion (Wrigley et al., 2009; Freund et al., 2011). The injury also results in deafferentation causing major rearrangement of the sensorimotor cortex (Nardone et al., 2013). Also, the physical disturbance causes a breakdown of the blood-brain barrier, permitting the entry of leukocytes. The secondary phase of neuronal injury includes demyelination, further disruption of synaptic connectivity, and increased oxidative stress, leading to increased cell death, fibroblast invasion, and inflammatory response. Eventually, a glial scar forms around the injury area which serves to wall off the injured area but also forms a barrier to axonal regeneration. There are also changes in the structure and composition of perineuronal nets after SCI, which could affect plasticity (Yi et al., 2012; Orlando and Raineteau, 2015). This failure of long-distance axonal regeneration and local plasticity are key features of the injury response and are major contributors to the lack of recovery of function. The exact cellular and molecular mechanisms that contribute to this failure are still a subject of active investigation but can be broadly classified into two distinct processes. The first is that adult neurons have limited capacity to grow due to changes in gene expression after development. The second is the inhibitory properties of the glial scar. Ultimately, combination therapies that overcome each of these deficits will be necessary.

Glial scar formation is a result of a series of pathological processes following traumatic injury. Injury to the brain typically produces vascular trauma, resulting in the entry of blood-borne cells (leukocytes and macrophages; Trivedi et al., 2006) as well as ischemia. This results in swelling and local edema (Mautes et al., 2000). Cells in the injured region release ATP which activates purinergic receptors on microglia to promote microglial chemotaxis in the lesion area (Davalos et al., 2005; Haynes et al., 2006; Bellver-Landete et al., 2019). Moreover, tissue reperfusion shortly after injury induces glutamate release resulting in excitotoxicity (Park et al., 2004) and oxidative stress and free radical release (Jia et al., 2012) resulting in the death of neurons and glia. Additionally, cellular and extracellular factors release DAMPs (damage-associated molecular patterns), which trigger the inflammatory response of stromal cells, astrocytes, oligodendrocyte progenitor cells (OPCs), and microglia (Pineau and Lacroix, 2007; Chen and Nuñez, 2010; Gaudet and Popovich, 2014; Didangelos et al., 2016). This cascade culminates in the development of the glial scar—a hallmark of the delayed phase of the injury response following spinal cord injury. While scars in other tissue generally resolve, the scars inside the central nervous system (CNS) are long-lasting (Camand et al., 2004). Though coined “glial scar” the lesion contains various cell types in addition to non-neural and extracellular components (Göritz et al., 2011; Silver et al., 2014).

Glial scars are made of two distinct parts—the lesion core and lesion border. Early on, microglia contribute to the formation of the scar through secretion of factors that activate astrocytes (Yang et al., 2020). In the mature glial scar, the lesion core consists of multiple cell types, including fibroblasts, macrophages, as well as NG2^+^ OPCs whereas the lesion border chiefly contains reactive astrocytes—characterized by changes in morphology and gene expression, high expression of intermediate filament proteins, and hypertrophy—NG2^+^ oligodendrocyte progenitor cells, and microglia (Ughrin et al., 2003; Busch et al., 2010; Cregg et al., 2014; Figure 1). The glial cells continue to secrete extracellular matrix (ECM) molecules, including tenascins and CSPGs, while fibroblasts secrete both CSPGs and collagens which form a dense matrix around the injury area (Wiese et al., 2012). Though glial scar formation is effective in enclosing the area of injury, reconstructing the damaged blood-brain barrier, and preserving any damaged tissue, it also inhibits axonal regeneration and outgrowth (Faulkner et al., 2004; Silver and Miller, 2004; Rolls et al., 2009). Major issues arise as to which of the properties of the scar are inhibiting plasticity and growth. Recent data support a protective role for astrocytes: if they are selectively ablated, there is significantly more inflammation and tissue damage (Faulkner et al., 2004). In contrast, increased numbers of macrophages populate the injury area and secrete inhibitory matrix molecules and promote axon dieback (Evans et al., 2014). As a result of restrictive ECM molecules, CSPGs, and a network of cells, the glial scar is also stiffer than uninjured tissue, which could provide a mechanical barrier to regenerating axons (Yu and Bellamkonda, 2001; Moeendarbary et al., 2017).

**Figure 1:**
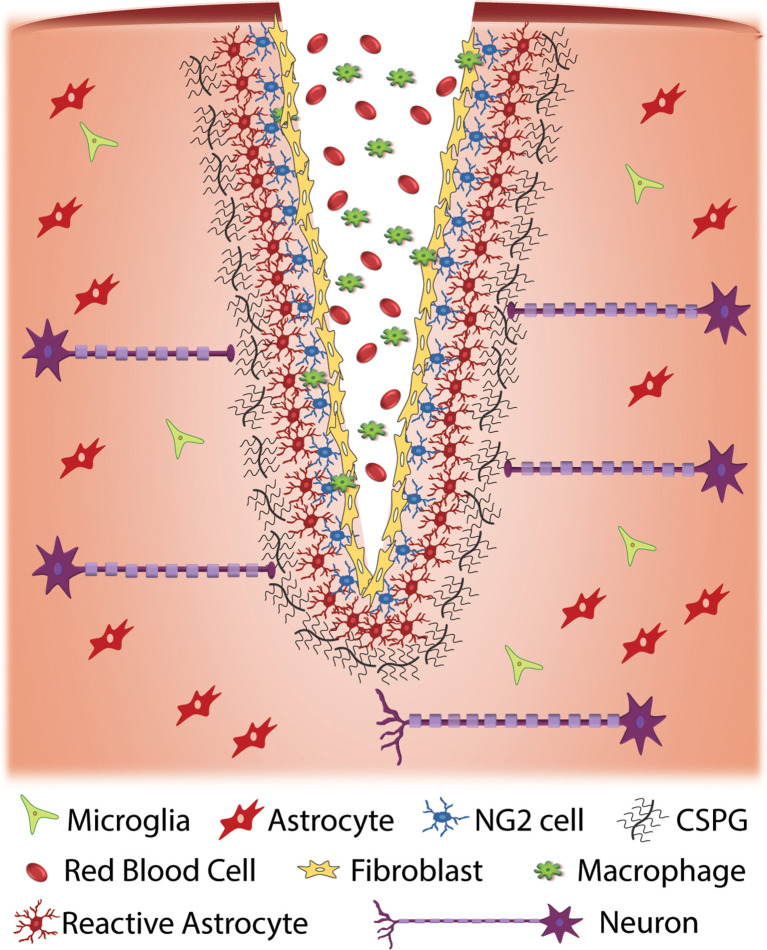
Cellular response to traumatic injury to the central nervous system (CNS). Injury to the CNS results in a series of events that result in the formation of a glial scar around the site of injury. Injury often results in the exposure of nervous system tissue to red blood cells and infiltration from macrophages and fibroblasts. Fibroblasts proliferate at the injury site and eventually form a fibrotic scar. Cellular damage due to injury and exposure to blood initiates an immune response that results in the activation of glial cells such as NG2 cells and astrocytes at the injury site. When activated, these cells produce extracellular matrix (ECM), specifically chondroitin sulfate proteoglycans (CSPGs), at an accelerated rate. The upregulation of CSPGs at the injury site is inhibitory to regenerative axons resulting in a cessation of axonal growth and the formation of retraction bulbs.

While the mixed cell populations of the glial scar contribute to scar formation, the glial scar environment is considered to be exceptionally inhibitory, primarily due to the presence of myelin-derived inhibitors as well as CSPGs. Macrophages, which are recruited to the area of injury, upregulate the levels of CSPGs. Following the initial studies in culture that demonstrated that axons of dorsal root ganglion cells turn at CSPG boundaries (Snow et al., 1990), many other studies have demonstrated that virtually all neuronal types respond to CSPG-rich regions *in vitro* or *in vivo* by turning (Höke and Silver, 1996).

CSPGs (Johnson-Green et al., 1991) consist of a core protein decorated by one or more CS GAG chains. Several different core proteins have been found in scar tissue, including aggrecan, neurocan, brevican and phosphacan (Yamada et al., 1997; Snow et al., 2001; Buss et al., 2009; Andrews et al., 2012). Of these, brevican seems to have the closest association with reactive astrocytes in the lesion core (Andrews et al., 2012; Pearson et al., 2020). While there is some evidence that these core proteins may signal to neurons (Iijima et al., 1991; Oohira et al., 1991; Ughrin et al., 2003), there is overwhelming evidence supporting the primary role of the GAG chains in providing inhibitory signals. The first evidence came from studies that demonstrated improved sprouting and recovery of function after the local application of the enzyme chondroitinase ABC, which digests the GAG chains, leaving the protein cores intact (Bradbury et al., 2002). Other studies have shown improved regeneration following the interruption of GAG chain synthesis by knockdown or knockout of the synthetic enzymes (Grimpe and Silver, 2004; Takeuchi et al., 2013).

The GAG chains of CS, dermatan sulfate (DS), and heparan sulfate (HS) are linear polysaccharides covalently attached to Ser residues in the various core proteins through a common GAG-protein linkage region of GlcUAβ1-3Galβ1-3Galβ1-4Xylβ1. The further polymerization of CS GAG chains is coordinated by six enzymes which are members of the chondroitin synthase family, including chondroitin synthases (ChSys; Kitagawa et al., 2001; Yada et al., 2003a,b), chondroitin-polymerizing factor (ChPF; Kitagawa et al., 2003), and CSGalNAcTs (Gotoh et al., 2002; Sato et al., 2003; Figure 2). These enzymes add the repeating disaccharide unit [-4-D-glucuronic acid (GlcA) β1-3 N-acetyl-D-galactosamine (GalNAc) β1-] to the chain. Following the addition of the disaccharide to the CS backbone, it is modified by the sulfation of hydroxyl groups at the C4 and C6 positions of GalNAc and C2 position of the GlcA residues. Each disaccharide may have a different combination of sulfations, and the nomenclature of disaccharide units with various modifications was proposed as shown in Supplementary Table S1 (Sugahara and Mikami, 2007).

**Figure 2:**
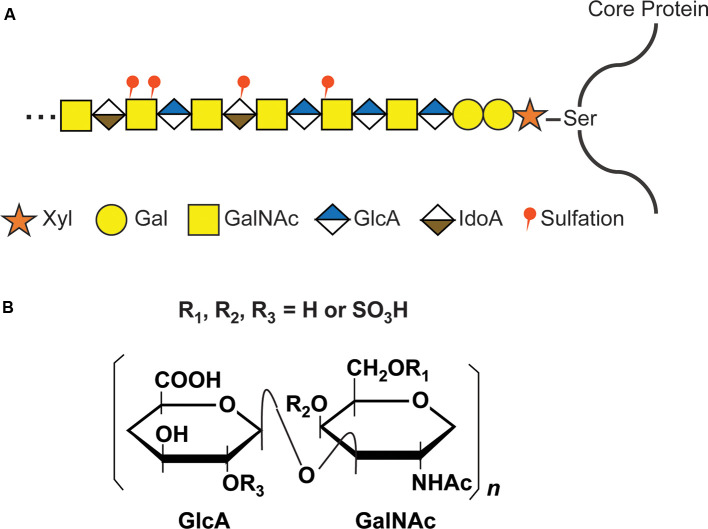
CS glycosaminoglycan (GAG) structure and modification by sulfation. **(A)** Schematic structure of CS GAG chains. CS-GAG chains are attached to the serine residues on the core protein *via* a tetrasaccharide linkage, followed by the addition of the repetitive disaccharide units modified by different sulfations. **(B)** Chemical structure of CS disaccharide. CS disaccharides are modified by sulfation at C-4 or C-6 position of GalNAC and/or C-2 position of GlcA.

The transfer of a sulfate group from the sulfate donor, 3′-phosphoadenosine 5′-phosphosulfate (PAPS) to the corresponding positions of GlcA and GalNAc is catalyzed by various sulfotransferases as shown in Figure 3 and Supplementary Table S2: the A unit, consisting of GlcA-GalNAc(4-*O*-sulfate), is mediated by chondroitin 4-*O*-sulfotransferases (C4ST-1, -2, -3, alternative names: CHST11, 12, 13; Okuda et al., 2000a,b; Yamauchi et al., 2000; Kang et al., 2001, 2002; Mikami et al., 2003). The C unit, consisting of GlcA-GalNAc(6-*O*-sulfate), is by chondroitin 6-*O*-sulfotransferase (C6ST-1, -2, alternative names: CHST3, 7; Fukuta et al., 1995, 1998; Mazany et al., 1998; Kitagawa et al., 2000). The E unit, consisting of GlcA-GalNAc(4,6-*O*-disulfate) is generated from the A unit by chondroitin 4,6-*O*-sulfotransferase (GalNAc4S-6ST, alternative name: CHST15; Ohtake et al., 2001, 2003), and the D-unit, consisting of GlcA(2-*O*-sulfate)-GalNAc(6-*O*-sulfate), is created by the addition of sulfate to the C unit by uronyl 2-*O*-sulfotransferase (UST; Kobayashi et al., 1999; Ohtake et al., 2005). Some of the GlcA residues in a chondroitin backbone are enzymatically epimerized at the C-5 position by dermatan sulfate epimerases (DSE, DSEL), resulting in the formation of L-iduronic acid (IdoA; Lindahl et al., 2015). A stereoisomer of the CS polysaccharide containing IdoA instead of GlcA residues is designated as DS (Maccarana et al., 2006; Pacheco et al., 2009). DS is often distributed as CS-DS hybrid GAG chains and sulfation of DS is mediated by dermatan 4-*O*-sulfotransferase (D4ST-1, alternative name: CHST14; Evers et al., 2001; Tykesson et al., 2018).

**Figure 3:**
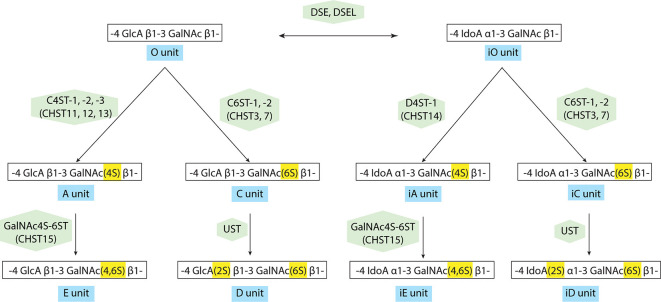
Modification pathway of CS/dermatan sulfate (DS) GAG chains. After the formation of CS GAG backbone, sulfotransferases transfer sulfate groups from 3′-phosphoadenosine 5′-phosphosulfate (PAPS) to the corresponding positions of GlcA and GalNAc. DS-epimerases convert GlcA into IdoA by epimerizing the C-5 carboxyl group in the chondroitin precursor, resulting in the formation of the dermatan backbone. D4ST1, distinct from C4ST, transfers a sulfate group from PAPS to the C-4 position of the GalNAc residues in dermatan to form the iA-units.

None of the processes for GAG chain synthesis or sulfation are template-driven, and each GAG chain may be unique in terms of its: (1) length; (2) degree of epimerization; and (3) degree of sulfation, resulting in immense heterogeneity. Because of the limited ability to sequence CS GAG chains, this large degree of variability within chains, coupled with both injured and uninjured situations in the complexity of the nervous system has resulted in confusing and often conflicting experimental results. The role of specific sulfation on GAG chains in the nervous system remains an area of interest in the field. It should be noted that the nomenclature of biologic CS GAG chains including CS-A, CS-C, CS-D, and CS-E is confusing since CS-A, for instance, is rich in A unit, a predominant CS isoform, but CS-A is indeed the mixture of A-, C-, and O-units. CS-C, CS-D, and CS-E are rich in C unit, D unit, and E unit, respectively. Various studies have revealed the structural diversity in vertebrate GAG chains attached to proteoglycans (Yamada et al., 2011) and CS GAG chains isolated from natural sources exist as a mixture of linear polysaccharides with different length and distinct patterns of sulfation.

While GAG chains are normally heterogeneous, the question has been posed as to whether chondroitin sulfate, like HS, may contain a “sulfation code” that harbors biological activity (Gama et al., 2006). In the context of nervous system regeneration, the experimental approach has focused on two aspects: (1) how neurons in culture react to GAG chains with different sulfation patterns; and (2) changes in GAG chain sulfation or amount after injury. In the *in vitro* experiments, the results are often inconsistent and can paint a confusing picture. As noted above, this may be due to the CSPGs utilized—natural products with heterogeneous composition—and neurons from different brain regions that may express different complements of receptors. For instance, some experiments showed that CS-A is inhibitory to cerebellar granule (Wang et al., 2008) and trigeminal (Schwend et al., 2012) neurites, but others showed that CS-A did not inhibit the cortical (Karumbaiah et al., 2011), dorsal root ganglion (Brown et al., 2012) or retinal (Shimbo et al., 2013) neurite growth. Other studies showed that CS-C inhibited the growth of neurites from cortical (Butterfield et al., 2010), retinal (Shimbo et al., 2013) and trigeminal (Schwend et al., 2012) neurons, while contradictory results were observed for the inhibitory effect on DRG neurons (Verna et al., 1989; Brown et al., 2012). No effect of CS-C on cerebellar neurites (Wang et al., 2008), and others found a similar lack of action on retinal (Shimbo et al., 2013) neurites. The oversulfated CS-D and CS-E have each been identified as having inhibitory or growth promotional actions. Cortical (Karumbaiah et al., 2011), retinal (Shimbo et al., 2013), and dorsal root ganglion (Brown et al., 2012) neurons were inhibited by CS-E. In contrast, hippocampal neurite outgrowth was generally promoted by CS-D and CS-E as well as several different oversulfated DS saccharides (Clement et al., 1998, 1999; Nadanaka et al., 1998; Hikino et al., 2003; Bao et al., 2004). These results appear to be inconsistent. As mentioned above, this could be in part because of the heterogeneity of CS GAG chains used. One approach is to use synthetic mimetics of CS GAG chains. There is a rich history in chemical synthesis of GAG chains (Jacquinet et al., 1998; Tamura and Tokuyoshi, 2004; Cai et al., 2012; Shioiri et al., 2016; Li et al., 2017; Zhang et al., 2020). However, there are significant issues that need to be addressed with their use. The conformation and presentation of GAG chains are important. Thus, a tetrasaccharide of CS-E was found to promote neuronal growth (Tully et al., 2004; Gama et al., 2006), yet a different CS-E polymer inhibited neurite outgrowth in culture (Rawat et al., 2008). It may be that growth promotion is through the interaction of highly sulfated CS GAGs with growth factors (Zou et al., 2003; Gama et al., 2006). Another issue is that large quantities of synthetic sugars are needed for biological experiments. Taken together, there is increasing evidence that the presence of certain sulfation moieties and the location of specific sulfation on a GAG chain may be critical to function in the nervous system, especially after an injury.

The role of 4-sulfation on CS GalNAc appears to be outsized in mediating the inhibitory actions of CS, as the 4-sulfation motif on GalNAc is intrinsic to both A and E units. In mammals, there is an increase in the ratio of 4-sulfation to 6-sulfation with aging, due not only to an increased level of 4-sulfated GAG chains but also a significant decrease in 6-sulfated GAG chains (Foscarin et al., 2017). This correlates with a loss of neural plasticity. In a tau model of neurodegeneration, plasticity was restored with an antibody against 4-sulfated GAG (Yang S. et al., 2017). Our investigation of changes in CS sulfation patterns following both spinal cord injury (Wang et al., 2008) and optic nerve crush (Pearson et al., 2018) have found a large increase in 4-sulfated GAG chains in the injury site, with little or no change in 6-sulfated GAG chains. In culture, the production of 4-sulfated, but not 6-sulfated GAG chains were increased in astrocytes after treatment with TGF-β (Wang et al., 2008). Also, an antibody against 4-sulfated GAG chains improved neurite outgrowth on aggrecan (Yang S. et al., 2017), as did the treatment of 4-sulfated GAG chains with chondro-4-sulfatase (Wang et al., 2008). Thus, it would seem that selectively targeting 4-sulfation on GAG chains might be an alternative strategy to the degradation of CS GAG chains with chondroitinase. The 4-sulfation motif is also prominent in zebrafish, and the reduction of 4-sulfation on CS by CHST11 knockdown improved regeneration in the animal (Sahu et al., 2019). While targeting biosynthesis of CS 4-sulfation should eliminate both CS-A and CS-E units, an alternative strategy is to target 4-sulfation with the use of specific enzymes. This has been accomplished with the enzyme, arylsulfatase B (ARSB). ARSB selectively removes 4-sulfation specifically at the non-reducing end of CS GAG chains (Matalon et al., 1974). In culture, ARSB was able to increase hippocampal neurite outgrowth on astrocytes treated with either TGF-β (Pearson et al., 2018) or ethanol (Zhang et al., 2014). This efficacy of ARSB has been confirmed *in vivo*, where it was able to improve locomotor function after spinal cord injury (Yoo et al., 2013), as well as optic nerve regeneration after a crush (Pearson et al., 2018). This provides some supporting evidence for a “sulfation code” by indicating that a specific sulfate at a specific location on a GAG chain is important for biological effects. Because ARSB is approved for the treatment of patients with Mucopolysaccharidosis type VI (Harmatz and Shediac, 2017), this selective approach to CS GAG chain modification needs to be tested in higher animals for equivalent actions.

The GAG chains of CS, DS, and HS interact with a variety of proteins to function in many physiological processes (Sarrazin et al., 2011; Mizumoto et al., 2015). The selectivity and specificity are not only dependent upon protein sequences but also the oligosaccharide sequences. Several studies have investigated the interactions of CS GAG chains with other molecules involved in neuronal growth and guidance. Semaphorin-3A (Sema3A), originally identified as collapsin-1 due to its ability to collapse growth cones (Luo et al., 1993), and 4-sulfated CS GAG chains were found to overlap in the striatum (Zimmer et al., 2010). Using an *in vitro* stripe assay, the combination of Sema3A and CSPGs potentiated the inhibitory actions of either (Zimmer et al., 2010). Sema3A was found to bind to CS-E in perineuronal nets (Dick et al., 2013), but another study from this same group showed that Sema3A binding to perineuronal nets was reduced by antibody Cat-316 (Lander et al., 1997), which binds to CS-A but not CS-E (Yang S. et al., 2017). Thus, whether Sema3A participates to restrict synaptic plasticity by perineuronal nets is still not clear. Sema5A was also reported to bind to both CS and HS GAG chains, leading to opposite actions: HS GAG chains seem to act cell-autonomously to promote axonal growth, while interaction with CS GAG chains in the ECM turns Sema5A into an inhibitory molecule (Kantor et al., 2004). In the cerebellum, D units of CS appear to mediate the binding of pleiotrophin in the control of dendritic growth (Shimazaki et al., 2005).

Accumulating evidence indicates the presence of functional receptors on the cell surface to transmit signals from GAG chains. As noted above, both CS-E and CS-D promote the outgrowth of hippocampal neurons. Contactin-1 was identified as a receptor involved in the promotion of neurite outgrowth by CS-E (Mikami et al., 2009). Surface plasmon resonance (SPR) was used to demonstrate a K_d_ in the micromolar range between CS-E and contactin-1, while CS-A/CS-C appeared not to interact with the molecule. Since contactin-1 is a GPI-anchored protein and lacks a cytoplasmic domain, it is likely to form a complex with other signaling molecules. The growth promotional actions of CS-D have been attributed to activating integrin α_V_β_3_ signaling with a micromolar K_d_ (Shida et al., 2019).

Two major classes of receptors have been identified that mediate the inhibitory actions of CS GAG chains on axonal growth. One is a subclass of the Receptor Protein Tyrosine Phosphatase (RPTP) type IIa members, consisting of Leukocyte common antigen-related phosphatase (LAR), RPTPσ and RPTPδ. All of the members contain extracellular immunoglobulin-like (Ig) domains and fibronectin III domains and cytoplasmic phosphatase domains (Stoker, 2001). LAR, which is widely expressed in various types of neurons in CNS, has been identified as a functional receptor for CSPGs (Fisher et al., 2011). A direct interaction was demonstrated by co-immunoprecipitation of LAR with CSPGs. This interaction leads to an inactivation of Akt and activation of Rho, resulting in axonal growth inhibition. LAR knockout mice or mice treated with LAR-targeting peptides showed an improved locomotor function after spinal cord injury (Fisher et al., 2011; Xu et al., 2015). RPTPσ, another member of the RPTP type IIa family, was also classified as a functional receptor (Shen et al., 2009). RPTPσ knockout mice displayed reduced CSPG inhibition after spinal cord injury and an enhanced regeneration after sciatic nerve crush injury, and RPTPσ-targeting peptides reduced the inhibitory action of CSPGs (Lang et al., 2015). The first two Ig domains of RPTPδ are close structural homologs of those of RPTPσ and contain an essentially identical GAG binding site (Coles et al., 2011). While direct evidence of CS GAG binding to RPTPδ is lacking in the literature, we have found this site was responsible for the binding for both heparin and CS-GAG (Katagiri et al., 2018).

Intriguing enough, binding of CSPGs to RPTPσ impedes axonal growth, whereas binding of heparan sulfate proteoglycans (HSPGs) to RPTPσ promotes the growth of axons, indicating RPTPσ as a bifunctional receptor in the regulation of neurite extension (Aricescu et al., 2002; Coles et al., 2011). Structural studies have demonstrated that the GAG-binding site for both CS and HS lies in the first Ig domain of RPTPσ. Biochemical analyses revealed that both CS-E and DS as well as heparin, a mimetic of HS, bound to RPTPσ with K_d_s in the nanomolar range (Katagiri et al., 2018). Phosphatase activity is essential for the biological effects of GAG binding to RPTPσ and LAR (Fisher et al., 2011; Lee et al., 2016), and several proteins, including cortactin, are substrates of RPTPσ (Sakamoto et al., 2019). A model was proposed where HS binding to the common GAG-binding site on RPTPσ induced clustering of the extracellular region of RPTPσ, whereas CS binding did not, and the opposing effects of HS and CS GAG chains were attributed to the differential oligomeric state of RPTPσ (Coles et al., 2011). More recently, we identified a novel binding site for HS in the juxtamembrane domain on RPTPσ, providing an additional mechanism through which RPTPσ is bifunctional (Katagiri et al., 2018; Figure 4). Further, a synthetic CS oligosaccharide was found to disrupt autophagy in a sulfation-dependent manner, leading to the inhibition of axonal regeneration (Sakamoto et al., 2019). Of particular interest is that presynaptically-expressed LAR and RPTPσ not only bind to GAG chains but also associate with postsynaptic binding partners that are involved in synaptic organization (Takahashi and Craig, 2013; Coles et al., 2014; Bomkamp et al., 2019). Thus, the interaction of RPTPs with GAG chains might regulate synapse development and/or plasticity as well as axonal growth.

**Figure 4:**
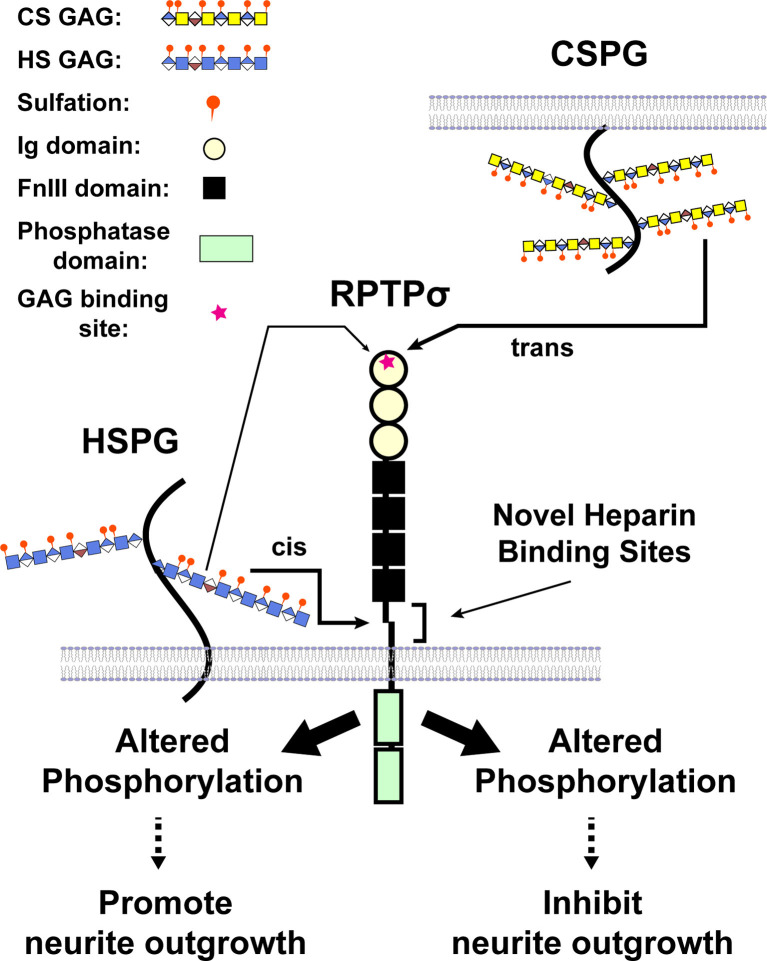
A model illustrating CS and heparan sulfate (HS) binding to RPTPσ. When CS/DS GAG chains are presented by different cells in trans, binding occurs through the first Ig domain far from the transmembrane domain and triggers the changes in phosphorylation (after Katagiri et al., 2018).

The other class of receptors are members of the Nogo receptor family, NgR1 (reticulon 4 receptor), and NgR3 (reticulon 4 receptor-like 1; Dickendesher et al., 2012). NgR1 was originally identified as a receptor for myelin-associated inhibitors (MAIs) including Nogo-A, myelin-associated glycoprotein (MAG), and oligodendrocyte myelin glycoprotein (Saha et al., 2014), while NgR3 did not interact with MAIs. Doubly sulfated CS-D and CS-E as well as DS were found to bind to NgR1 and NgR3 with nanomolar K_d_. Mice lacking both NgR1 and 3 showed increased regeneration after injury (Dickendesher et al., 2012). Similar to contactin-1, NgR1 and NgR3 are GPI-anchored proteins, indicating the importance of signaling complex with these molecules, and engagement of CSPGs and NgR1 induced the activation of RhoA (Saha et al., 2014).

As noted above, 4-sulfated CS is associated with inhibition of axonal outgrowth (Wang et al., 2008; Yoo et al., 2013; Pearson et al., 2018). Both CS-E and DS, which bind to both RPTPσ and NgR1 and 3, contain 4-S GalNAc, but how 4-sulfation affects binding of these sugars to these receptors is yet to be determined. Interestingly, CS-A, which is rich in 4-S GalNAc, does not bind to either receptor (Dickendesher et al., 2012), suggesting that there may be another receptor for 4-S GAG. A series of peptides that bind to 4S-GAG have differential actions on several neuronal properties, including neurite outgrowth, providing further evidence for additional receptors for 4-S GAG (Loers et al., 2019). Also, it would be interesting to know if these peptides disrupt the binding of CS to their known receptors.

The signaling pathways activated by CS GAGs also provide potential therapeutic targets. Neurons growing on CSPG substrates or exposed to CSPGs show an increase of active RhoA (Borisoff et al., 2003; Fisher et al., 2011), and many experiments have demonstrated that inhibition of RhoA or Rho-kinase can increase axonal growth, both in the presence of CS *in vitro* (Dergham et al., 2002; Borisoff et al., 2003) or *in vivo* (Sellés-Navarro et al., 2001). This increase in RhoA was not observed in neurons with a deletion of LAR (Fisher et al., 2011). Similarly, the treatment of cerebellar granule neurons with CSPG increased active RhoA, but this was not observed in CGNs with a deletion of RPTPσ (Ohtake et al., 2016). Interestingly, other treatments that increase neurite outgrowth in response to CSPGs, such as non-steroidal anti-inflammatory agents (Fu et al., 2007), reduce RhoA activation. Active RhoA inhibits myosin light chain phosphatase, ultimately altering myosin II activity, and inhibiting myosin II with blebbistatin can overcome CSPG inhibition (Hur et al., 2011; Yu et al., 2012). A reduction in Akt activity is seen in parallel with the increase in RhoA (Fisher et al., 2011). Growth cone contact with CSPGs induces an influx of extracellular Ca^2+^ (Letourneau et al., 1994), and blocking L-type Ca^2+^ channels can promote neurite outgrowth on CSPGs (Huebner et al., 2019). CSPG also activates PKC in neurons (Sivasankaran et al., 2004), and downregulation or blockade of PKC can overcome CSPG inhibition (Powell et al., 1997; Sivasankaran et al., 2004). While the exact isoform of PKC responsible for inhibition has not been determined, it is likely a conventional (Ca^2+^-sensitive) one. Interestingly, inhibition of PKC activity also reduced activation of RhoA in response to CSPGs (Sivasankaran et al., 2004). Several other signaling pathways, including the PI3Kinase/AKT (Fisher et al., 2011), MAP Kinase (Kaneko et al., 2007), EGF receptor (Koprivica et al., 2005), and HDAC6 (Rivieccio et al., 2009) have also been implicated in CSPG signaling, but the exact function of these signaling pathways and how they interact is still an open question.

Our understanding of how GAG chains and other ligands interact with these receptors, as well as their downstream signaling, is still limited, in no small part due to the absence of reagents. Further advances in this field will depend upon improvements in both synthetic and analytical techniques. Thus, much of the current confusion about the role of sulfation is due to the dependence on heterogeneous GAG chains obtained from biological sources; synthesis of homogeneous GAG chains should provide a significant advantage to these studies. One such approach is glycoarray technology. Libraries generated from chemically-synthesized as well as natural glycans are immobilized and permit the analysis of protein-carbohydrate interactions (Puvirajesinghe and Turnbull, 2016; Ricard-Blum and Lisacek, 2017; Yang J. et al., 2017; Pomin and Wang, 2018). Another approach is the creation of stable cell variants with CRISPR-Cas9 technology that produce divergent and novel GAG structures (Chen et al., 2018; Qiu et al., 2018). Binding experiments with these cell libraries showed that structure-activity relationships correlate to GAG fine structure. Also, the ability to sequence GAG chains should provide critical insight into structure-function relationships. Progress in mass spectrometry-based glycomics in the past decades has been exciting with the aid of bioinformatics (Ricard-Blum and Lisacek, 2017; Sethi et al., 2020). Ultimately, glycosamino-glycomics would require the capability of unbiased sequencing of any oligosaccharides derived preferentially from the natural source. Finally, understanding how GAG chain composition and receptor expression are regulated in different populations of neurons during normal development and after an injury is needed.

Traumatic spinal cord injury presents a unique challenge considering the devastating long-term neurological impact it produces. While recent research has advanced our understanding of the inhibitory cues and the role of specifically sulfated CS GAGs in this inhibition, many challenges remain as we seek to move into more translatable therapeutics. Approaches that employ multiple strategies could maximize functional neuronal recovery with axons that can feasibly regenerate and connect to their targets making combinatorial therapies an area of emphasis in future research. Determining which factors are key to optimal levels of regeneration, as well as ways to stimulate a wider population of neurons into a growth state, will be critical steps as we aim to fully repair adult CNS injury.

## Author Contributions

Each author contributed to the writing and editing of this manuscript.

## Conflict of Interest

The authors declare that the research was conducted in the absence of any commercial or financial relationships that could be construed as a potential conflict of interest.

## Supplementary Material

TABLE S1 Nomenclature of CS/DS disaccharide units.

TABLE S2 Human Chondroitin Sulfotransferases.
