# Supplementary material for: Role of Chondroitin Sulfation Following Spinal Cord Injury
Source: Front Cell Neurosci. 2020 Aug 5;14:208. doi: 10.3389/fncel.2020.00208 (PMC7419623; doi:10.3389/fncel.2020.00208)
Supplement: TABLE S1 — Nomenclature of CS/DS disaccharide units. [file Table_1.DOCX]

| **Table 1. Typical disaccharide units in CS and DS** | | |
| --- | --- | --- |
|  |  |  |
| **Symbol** | **Sequence** | |
| O unit | GlcA-GalNAc | |
| A unit | GlcA-GalNAc(4S) | |
| C unit | GlcA-GalNAc(6S) | |
| D unit | GlcA(2S)-GalNAc(6S) | |
| E unit | GlcA-GalNAc(4S, 6S) | |
| iO unit | IdoA-GalNAc | |
| iA unit | IdoA-GalNAc(4S) | |
| iC unit | IdoA-GalNAc(6S) | |
| iD unit | IdoA(2S)-GalNAc(6S) | |
| iE unit | IdoA-GalNAc(4S, 6S) | |
