# Supplementary material for: Role of Chondroitin Sulfation Following Spinal Cord Injury
Source: Front Cell Neurosci. 2020 Aug 5;14:208. doi: 10.3389/fncel.2020.00208 (PMC7419623; doi:10.3389/fncel.2020.00208)
Supplement: TABLE S2 — Human Chondroitin Sulfotransferases. [file Table_2.DOCX]

| **Table 2. Human Chondroitin Sulfotransferases** | | | |
| --- | --- | --- | --- |
| **Enzyme name** | **Coding gene** | | **Accession number (Human)** |
|  |  | alternative name |  |
| Chondroitin 4-O-sulfotransferase | C4ST-1 | Chst11 | NM_018413, NM_001173982 |
|  | C4ST-2 | Chst12 | NM_001243794, NM_001243795, NM_018641 |
|  | C4ST-3 | Chst13 | NM_152889 |
| Chondroitin 6-O-sulfotransferase | C6ST-1 | Chst3 | NM_004273 |
|  | C6ST-2 | Chst7 | NM_019886 |
| Uronyl 2-Sulfotransferase | UST |  | NM_005715 |
| Dermatan 4-O-sulfotransferase | D4ST-1 | Chst14 | NM_130468 |
| N-Acetylgalactosamine 4-Sulfate 6-O-Sulfotransferase | GalNAc4S-6ST | Chst15 | NM_015892, NM_014863, NM_001270764, NM_001270765 |
| Dermatan sulfate epimerase | DSE | Sart2 | NM_013352, NM_001080976, NM_001322937, NM_001322938 |
|  |  |  | NM_001322939, NM_001322940, NM_001322941, NM_001322944 |
|  |  |  | NM_001322943, NM_001374520, NM_001374521, NM_001374522 |
| Dermatan Sulfate Epimerase Like | DSEL |  | NM_032160 |
